# Enhanced High-Temperature Mechanical Properties of Al–Cu–Li Alloy through T1 Coarsening Inhibition and Ce-Containing Intermetallic Refinement

**DOI:** 10.3390/ma12091521

**Published:** 2019-05-09

**Authors:** Xinxiang Yu, Zhiguo Zhao, Dandan Shi, Han Dai, Jie Sun, Xiaoyan Dong

**Affiliations:** 1 Laboratory of Advanced Light Alloy Materials and Devices, Yantai Nanshan University, Longkou 265713, China; daihan1985@189.cn (H.D.); sunjie19860304@163.com (J.S.); pigeon929@126.com (X.D.); 2 Postdoctoral Station of Nanshan Group Co., Ltd., Longkou 265706, China; 3 Testing Center, Hang Xin Material Technology Co., Ltd., Longkou 264006, China; zhaozhiguo@nanshan.com.cn (Z.Z.); shidandan@nanshan.com.cn (D.S.)

**Keywords:** Al–Cu–Li alloy, cerium, thermal stability, intermetallic, microstructure

## Abstract

The effects of the addition of 0.29 wt% Ce on the high-temperature mechanical properties of an Al–Cu–Li alloy were investigated. Ce addition contributes to T1 (Al_2_CuLi) phase coarsening inhibition and Ce-containing intermetallic refinement which greatly improved the thermal stability and high-temperature deformation uniformity of this alloy. On the one hand, small Ce in solid solution and segregation at phase interface can effectively prevent the diffusion and convergence of the main element Cu on T1 phase during thermal exposure. Therefore, the thermal stability of Ce-containing alloy substantiality improves during thermal exposure at the medium-high-temperature stage (170 °C to 270 °C). On the other hand, the increment of the tensile elongation in Ce-containing alloy is much greater than that in Ce-free alloy at high temperatures tensile test, because the refined Al_8_Cu_4_Ce intermetallic phase with high-temperature stability are mainly located in the fracture area with plastic fracture characteristics. This work provides a new method for enhancing high-temperature mechanical properties of Al–Cu–Li alloy which could be used as a construction material for high-temperature structural components.

## 1. Introduction

Al–Cu–Li alloy has a number of advantages over conventional aluminum alloy, such as lower density, higher strength, higher fracture toughness, better fatigue, and corrosion resistance [1,2]. These properties contribute to the improvement of payload and fuel efficiency, thereby having made Al–Cu–Li alloy a competitive alternative to conventional 2xxx and 7xxx series materials in new aircraft designs [3]. The evaluation of thermal stability of Al–Cu–Li alloy is urgently needed for the potential application on fuselages, wing structures, bulkheads near the engine, as well as other high-temperature structural components which require high-temperature resistance in modern fighter aircraft [4]. So far the lower exposure temperatures in the range of 343 K to 358 K (70 °C to 85 °C) have been chosen to simulate the environment to which the wings and fuselage structures of commercial aircraft are exposed [5]. Reports have also been made on thermal exposure of Al–Cu–Li alloys at medium-high-temperatures (below 200 °C) [6,7]. However, materials near the engine, like bulkheads, are usually exposed to high-temperature environments (>200 °C). Therefore, the changes of microstructure and performance of Al–Cu–Li alloy in this temperature range requires further clarification, yet it has seldom been reported.

The high-temperature (>70 °C) mechanical properties of Al–Cu–Li alloy depends on the thermal stability of its precipitates which includes T1 (Al_2_CuLi), δ′ (Al_3_Li), and θ′ (Al_2_Cu) [8]. Among them, the T1 phase is the most stable [9], as the plates of T1 are very thin (~1.3 nm) and their thickness is very stable with aging at temperatures below ~170 °C [2]. Therefore, we chose T1 as the dominant strengthening phase in Al–Cu–Li alloy. The T1 phase can be promoted by increasing Cu/Li ratio [10] and small pre-stretching prior to artificial aging [2,11]. In addition, the thermal stability of T1, a Cu-containing precipitate, can be further improved by adding rare elements. A small addition of Sc [12], Y [13], La [14], and Er [15] can decrease the diffusion rate of the main alloying element Cu, thereby delaying the coarsening of Cu-containing precipitates in Al alloy. Ce as rare earth element also has the same function [16,17], so the coarsening process of the Cu-containing precipitate in Ce-containing alloy at high temperature can be delayed [18] and the thermal stability of these alloys can be improved, e.g., the addition of Ce inhibits the coarsening of Ω (Al_2_Cu) precipitate in Al–Cu–Mg–Ag alloy [19] and T1 (Al_2_CuLi) precipitate in Al–Cu–Li alloy [20]. Ce can also contribute to the refinement of intermetallic of aluminum alloys, e.g., a refined eutectic structure in 7055Al alloy [21], Al–6.7Zn–2.6Mg–2.6Cu (wt %) alloy [22], and Al–Cu–Li alloy [23] have been reported. A large number of small Al_8_Cu_4_Ce phases were also formed in Ce-containing Al–Cu–Li alloy after homogenization in our previous work [24]. These high melting point intermetallics do not coarsen evidently at elevated temperatures [25], which might strengthen grain boundaries and serve as obstacles to slip and creep at high temperatures to improve high-temperature mechanical properties of Ce-containing Al–Cu–Li alloy. Refined Ce-containing intermetallic improves the high-temperature mechanical properties of the Sn–3.8Ag–0.7Cu solder joint [26], but research on the Al–Cu–Li alloy from this approach has seldom been made.

The purpose of this work is to investigate the effect of Ce addition on the inhibition of the main strength phase T1 coarsening during high-temperature exposure in high Cu/Li ratio Al–Cu–Li alloy at T8 temper (6% pre-stretching), and the effect of Ce on the diffusion of Cu in this alloy by a designed diffusion couple. Moreover, the enhanced deformation uniformity and strength of the Al–Cu–Li alloy owing to the refined Ce-containing intermetallic during high-temperature tensile were also studied in this paper.

## 2. Materials and Methods

The materials used in this study were a 0.29 wt% Ce-containing alloy and a Ce-free Al–Cu–Li alloy, as shown in Table 1. Master alloys of Al–Cu (50 wt%), Al–Zr (3.29 wt%), Al–Ce (10 wt%), and pure Ag, Mg, Li, and the remaining Al were melted in a vacuum induction melting furnace in a controlled Ar gas atmosphere, in a high-purity graphite crucible [1]. The casting was performed in Ar, using a Cu mold surrounded by cooling water [27]. The ingots were homogenized through a two-step homogenization course at 470 °C, 8 h and 510 °C, 16 h in a salt bath, followed by air cooling. After that, the ingots were hot-rolled from 23 mm to 4.5 mm and finally cold-rolled to 2.2 mm in thickness. Subsequently, the plates were solution-treated at 520 °C for 1 h, water-quenched, and finally aged at 150 °C with pre-stretching at 6% (T8 temper).

In order to assess the thermal stability of the experimental alloys during thermal exposure, several specimens 10 mm × 15 mm × 2 mm in size, were cut from the plate at T8 temper. These specimens were then subjected to different over-aging heat treatments within the temperature range of 70 °C–320 °C, the soaking time ranging from 250 h up to 1300 h. The microhardness was measured by HV-1000 (Shanghai Yanrun Light Machinery Technology Co., Ltd., Shanghai, China), with a loading force of 0.2 kg and a loading time of 20 s. Tensile tests of specimens were performed at the temperatures from 25 °C to 300 °C, an Instron machine (INSTRON, Boston, MA, USA) equipped with incubator was used. The specimens were taken in the longitudinal direction from alloy plates at T8 temper.

The microstructure of the precipitate was examined by transmission electron microscopy (JEOL-2100F, JEOL Corp., Tokyo, Japan) at an accelerating voltage of 200 kV. TEM foils were prepared by the twin–jet polishing technique using a solution of 25% nitric acid and 75% methanol at −30 °C, 70–80 mA, and 15 V. Grain structure of the specimens was determined after being anodized with Barker’s reagent (1.8% HBF_4_ solution with a voltage of 20–30 V and a current of 0.5–1.5 mA, Quanzhou, China) and viewed with polarized light on optical microscope (ZEISS, Oberkochen, Germany). The evaluation of the fracture features of the high-temperature tensile specimen was performed with an FEI-Nova Nano SEM 450 scanning electron microscope (FEI Company, Hillsboro, OR, USA).

To verify the inhibiting effect of small Ce addition on T1 phase coarsening, a diffusion couple was designed. Firstly, a pure aluminum plate was sandwiched between a Ce-free alloy and a Ce-containing alloy (The oxide layer at the joint of alloy sheet was polished clean by water grinding sand). Then it was drilled and fixed with copper wire. After short-time preheating, it was tightly welded by rolling and then annealed at 520 °C for 6 h. The formation of a new oxide layer was prevented by inert gas protection during annealing and rolling. The cross section of the specimen was polished in accordance with metallographic standard and then observed by SEM.

## 3. Results and Discussion

**Figure 1:**
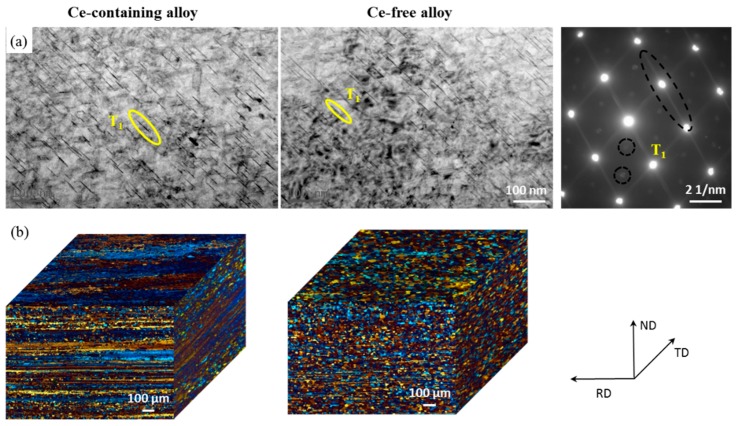
Microstructures comparison of Ce-containing and Ce-free alloys at T8 temper: (**a**) TEM image of T1 phase; (**b**) three direction optical micrographs of grain structure.

The initial microstructures of Ce-containing and Ce-free alloys at T8 temper were characterized both by TEM and three-dimensional OM investigations, as shown in Figure 1. Figure 1a shows that the main precipitate in two alloys is uniformly distributed T1 phase at T8 temper. A small pre-stretching before aging induces dislocation network within the matrix, which acts as heterogeneous nucleation sites for the T1 phase and greatly promotes T1 phase precipitation [28]. 

Although the distribution characteristics of T1 phase are not affected by Ce addition, many fiber-like unrecrystallized microstructures are found distributed along the rolling direction in the Ce-containing alloy while the recrystallized grains are primarily observed in Ce-free alloy (Figure 1b), Clearly Ce addition effectively improves the recrystallization resistance of Al–Cu–Li alloys during solid solution treatment [1]. Therefore, the initial microhardness of Ce-containing alloy (189 HV_0.2_) is greater than that of Ce-free alloy (177 HV_0.2_). Moreover, this is mainly attributed to the grain size difference between them.

**Figure 2:**
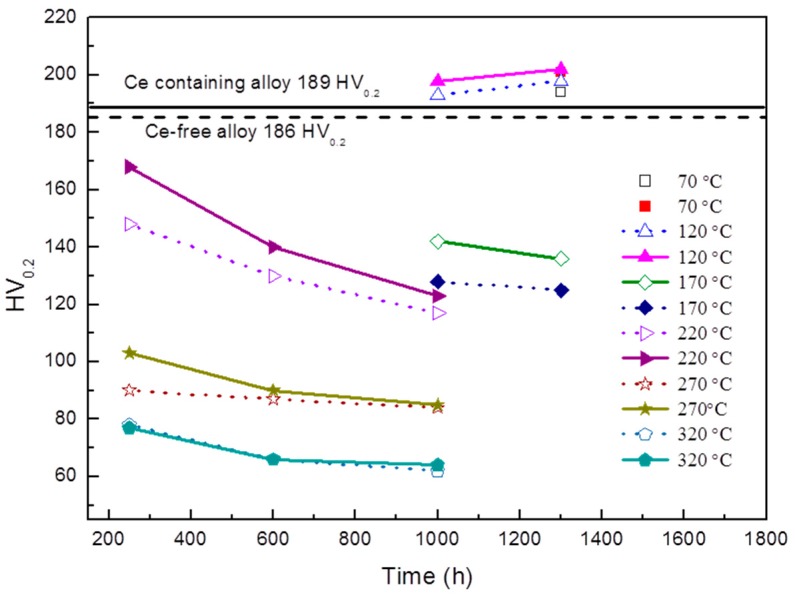
Thermal stability curves of Ce-containing and Ce-free alloys during thermal exposure at the temperature range 70–320 °C for soaking time from 250 h to 1300 h.

The thermal stability curves of Ce-containing and Ce-free alloys at T8 temper which are characterized by microhardness and obtained in the range 70–320 °C for soaking time from 250 h to 1300 h, are reported in Figure 2. The microhardness of Ce-containing alloy is found to be much higher than that of Ce-free alloy within the temperature range from 170 °C to 270 °C. At temperatures no higher than 120 °C, good thermal stability is observed in both alloys. As the temperature further increases to 320 °C, the hardness values of the two alloys become similar again. The thermal stability of the Al–Cu–Li alloy is substantially improved by small Ce addition during thermal exposure at medium-high-temperature stage (170 °C to 270 °C).

**Figure 3:**
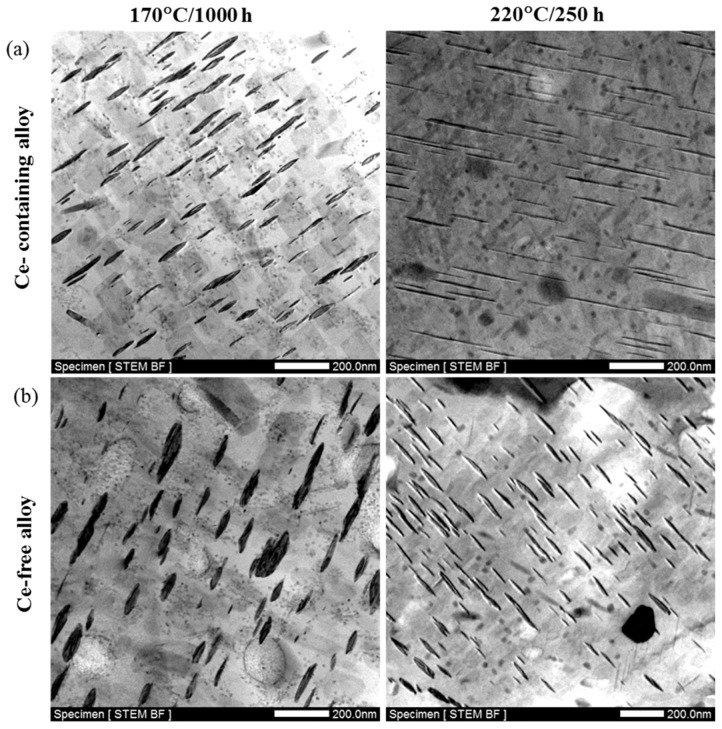
The precipitate phase coarsening characteristics of (**a**) Ce-containing and (**b**) Ce-free alloys after exposure at 170 °C for 1000 h and 250 °C for 250 h.

The precipitate phase characteristics of Ce-containing and Ce-free alloys after thermal exposure at 170 °C for 1000 h and 220 °C for 250 h were investigated respectively by using TEM, as shown in Figure 3. The thickness of the precipitates in Ce-containing alloy is 25.40 ± 6.52 nm (average ± standard deviation), which is much lower than that in Ce-free alloy (31.22 ± 10 nm) after exposure at 170 °C for 1000 h. Although the diameter (length) of the precipitates in Ce-free alloy (114.45 ± 29.70 nm) is slightly more than that in Ce-containing alloy (106.35 nm ± 28.19 nm), the effect of thickness increment of the precipitates (22.91%) is much more potent compared with that of their diameter increment (7.62%). After exposure at 220 °C for 250 h, the thickness of precipitates in Ce-containing alloy becomes 16.85 ± 3.41 nm, which is much lower than that in Ce-free alloy (27.58 ± 7.25 nm). Moreover, the diameter of precipitates in Ce-containing alloy turns to 196.11 ± 55.65 nm, which is much greater than that in Ce-free alloy (113.46 ± 35.96 nm). Ce addition is beneficial to slowing down the coarsening rate of the main phase T1 in the Al–Cu–Li alloy. These plate phase T1 precipitate along the {111}_α_ and it is non-deformable [29], the “strengthening stress *τ_p_*” produced by these phases can be expressed as [30].

(1) τp=0.12Gb(Dt)1/2[fν12+0.70(D/t)12fν+0.12(D/t)fν32]ln0.079Dr0

**Figure 4:**
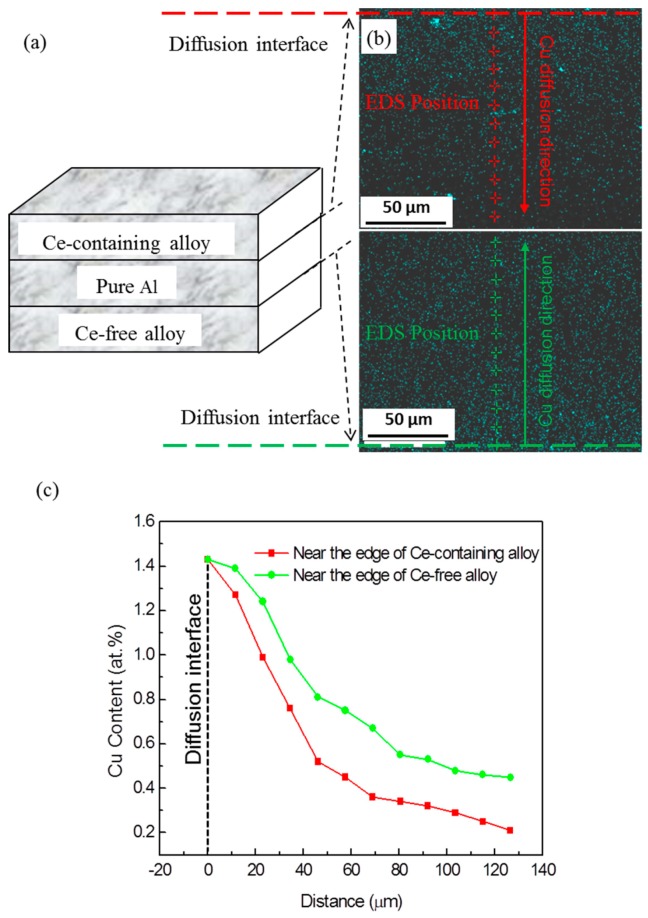
(**a**) Diagram of diffuse couple model; (**b**) mapping of Cu distribution and (**c**) Cu content distribution curve along the solid line arrow in pure Al near the edge of the Ce-containing alloy and the Ce-free alloy respectively.

*D* and *t* refer to the diameter and thickness of T1 phase respectively. Formula (1) shows that the strengthening stress varies inversely with the thickness and directly with the diameter of the T1 phase. Because Ce addition is beneficial to slowing down the coarsening rate of the T1 phase in the Al–Cu–Li alloy, the thickness of the precipitates of T1 in the Ce-containing alloy is much lower than that in Ce-free alloy during thermal exposure at 170 °C for 1000 h and 220 °C for 250 h. Since Cu is one of the main elements in the T1 phase, the coarsening rate of the main strengthening phase T1 in the Al–Cu–Li alloy can be reduced through inhibiting the diffusion and convergence of the main element Cu on T1 phase during medium-high temperature thermal exposure. To prove this inference, a diffusion couple was designed to evaluate the effect of Ce addition on the diffusion of Cu in the Al–Cu–Li alloys, as shown in Figure 4. Two diffusion interfaces were taken as the starting point, and a quantitative EDS (Energy scattering X-ray spectrum) analysis was done along the solid line arrow in every 10 μm in Figure 4. The diffusion rate of Cu atoms from Ce-containing alloy to pure Al is much lower than that from Ce-free alloy to pure Al, as shown in the mapping of Cu distribution and Cu content distribution curve along the solid line arrow. So the reduced diffusion rate of Cu in the Al–Cu–Li alloy through Ce addition was confirmed by the diffusion couple experiment.

The effect of Ce addition is attributed to the strong interaction energy between rare earth Ce and vacancies in solid solution matrix, which can prevent the migration of dislocation and solute atom [16,17]. Hence small Ce in solid solution can prevent the diffusion of major element Cu in experimental alloys, and ultimately inhibit the coarsening of strengthening phases T1 in the Al–Cu–Li alloy. Furthermore, due to the large size mismatch of Ce and Al atoms, Ce tends to segregate at the crystallographic interfaces for relieved lattice strain. The segregation of Ce at the edge interface of precipitates would effectively restrict its lateral growth and inhibit its coarsening. This would likewise decrease the copper diffusion rates as it decreases the tendency for higher diffusive flux between precipitates looking to coarsen. Therefore, the improved thermal stability of the Al–Cu–Li alloy during thermal exposure at the medium-high-temperature stage is attributed to the coarsening inhibition of the main strengthening phases T1 by reducing the diffusion rate of Cu element and restricting lateral growth through small Ce addition.

**Figure 5:**
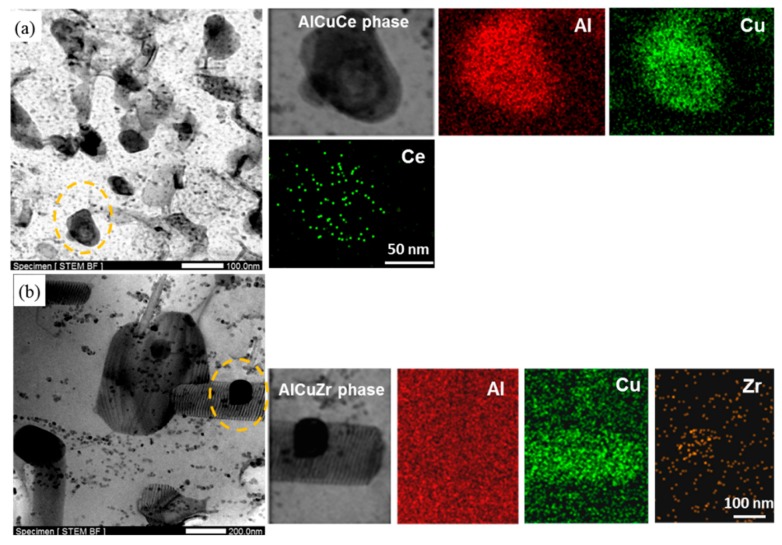
The characteristics and STEM-EDS mapping analysis of precipitates in (**a**) Ce-containing and (**b**) Ce-free alloys after exposure at 270 °C for 250 h.

Figure 5 is the microstructure and element mapping of precipitates in Ce-containing and Ce-free alloys after exposed at 270 °C for 250 h. Although the main strength phase T1 has been completely dissolved, a few new particles containing Al, Cu, and Ce with an average size of about 80 nm were found dispersed in the matrix. Moreover, a large number of smaller and uniformly distributed particles in the matrix were also observed in Ce-containing alloy, as shown in Figure 5a. In contrast to the case for Ce-containing alloy, some coarse rod-like phases with “fingerprint patterns” can still be seen in Ce-free alloy, which are dissolution marks of the residual phases of severely coarsened T1 phase. Only very few coarse AlCuZr particles with the size of 200 nm, and some small unevenly distributed particles can be seen in Ce-free alloy, as shown in Figure 5b. The hardness difference between the two alloys is about 15 HV_0.2_ (strength value is about 50 MPa), which is basically due to the contribution of the newly formed AlCuCe phase in Ce-containing alloy. Since the AlCuCe particles and those smaller newly observed particles are evenly dispersed in the alloy and remain stable in high temperature, Ce-containing alloy still exhibits better thermal stability than Ce-free alloy after thermal exposure at 270 °C for 250 h.

**Figure 6:**
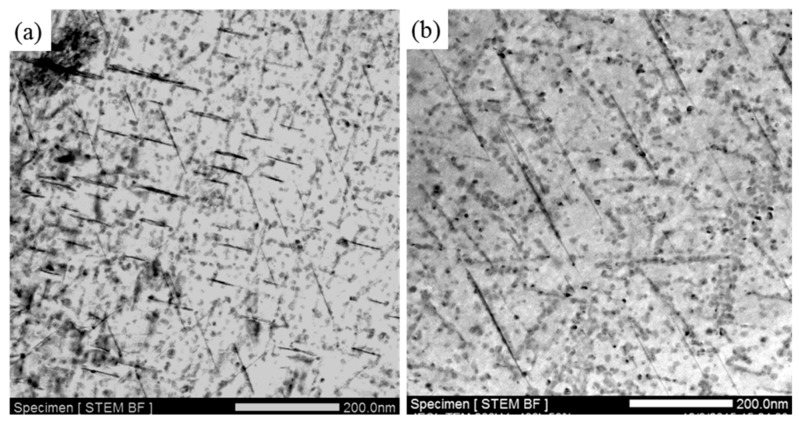
The precipitate characteristics of (**a**) Ce-containing and (**b**) Ce-free alloys after exposure at 120 °C for 1300 h.

**Figure 7:**
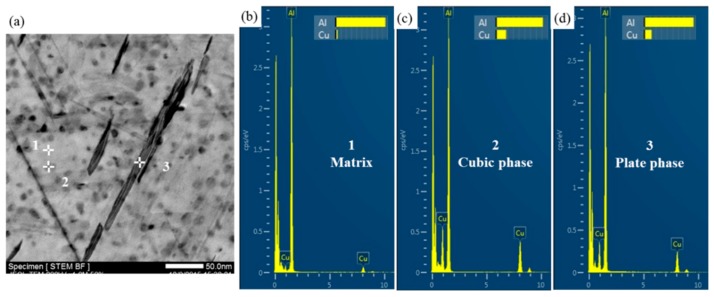
(**a**) TEM image of the precipitates in Ce-free alloy after exposure at 120 °C for 1300 h; STEM-EDS analysis of the matrix (**b**), cubic phase (**c**), and plate phase (**d**) as point 1, point 2, and point 3, respectively.

In addition, the two experimental alloys exhibit excellent thermal stability during exposure at temperatures below 120 °C, as shown in Figure 2. So the microstructures of the alloys after exposure at 120 °C for 1300 h were also observed by TEM, as shown in Figure 6. The stable strengthening phase T1 of the Al–Cu–Li alloy does not coarsen in both experimental alloys and a large number of densely distributed particles appear in the matrix, this is the main reason why the thermal stability of these alloys is maintained during exposure at 120 °C. The relative Cu content of the newly formed particle is compared semi-quantitatively with that of the matrix and plate phase T1 by STEM-EDS, as shown in Figure 7. The Cu content of this new particle is higher than that of T1 phase, thus the particle might be the square-shaped Al_5_Cu_6_Li_2_ phase [31]. The Al–Cu–Li alloys exhibit excellent thermal stability during exposure at temperatures below 120 °C because there is no obvious coarsening of the stable T1 phase and the fine Al_5_Cu_6_Li_2_ phase is formed at the same time.

**Figure 8:**
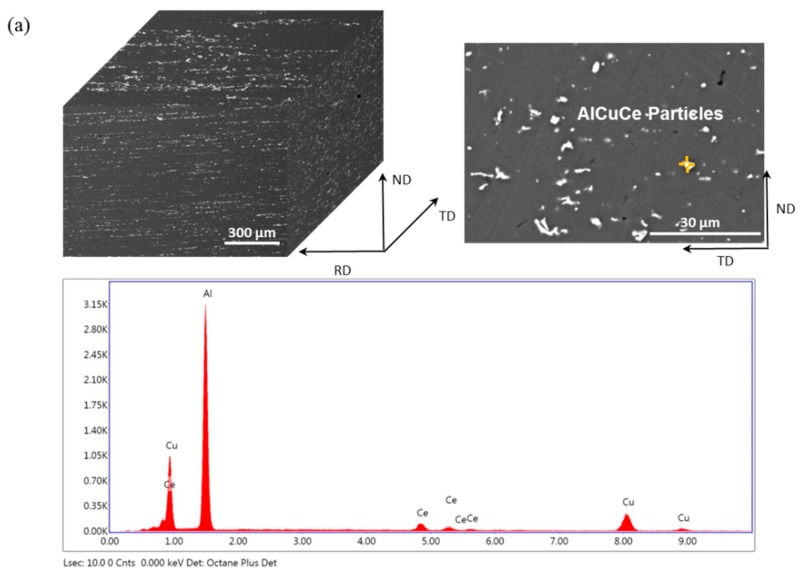

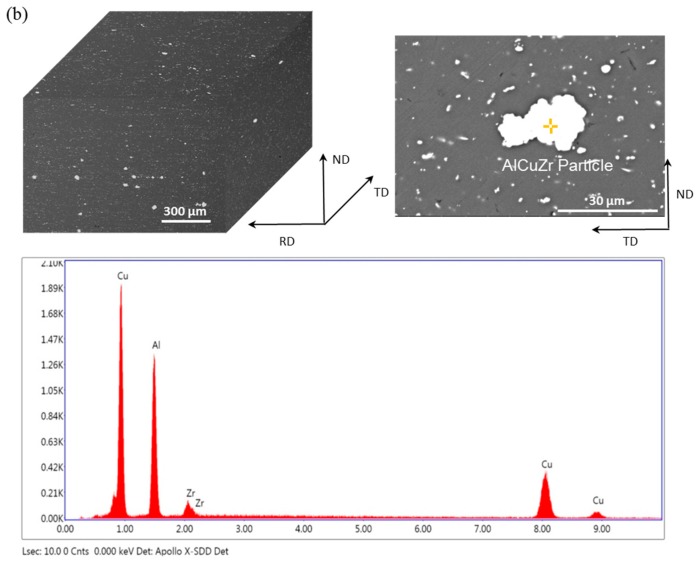
The characteristics and EDS analysis of the intermetallic in (**a**) Ce-containing and (**b**) Ce-free alloys after solid solution at 520 °C for 1 h.

The size and morphology of intermetallics in Ce-containing and Ce-free alloys after solid solution were investigated by SEM and EDS, as shown in Figure 8. The average transverse size of intermetallic particles AlCuCe in Ce-containing alloy is less than 6 μm. However, a few coarse intermetallic particles AlCuZr about 25 μm are also observed besides the normal small particles in Ce-free alloy. Obviously, the formation of coarse AlCuZr intermetallic compounds was interrupted by the strong attraction between Cu and Ce atoms in Ce-containing alloy [32], for the electronegative differences between Ce and Cu are more than that between Zr and Cu [17]. In addition, Ce and Zr have the opposite microsegregation patterns similar to that of Mn and Zr in Al alloy during solidification [33], so the Ce and Zr atoms combine with Cu respectively and transport in the opposite direction during solidification, thereby avoiding the formation of coarse AlCuZr phase. Moreover, the Ce addition would affect and refine the formation of interdendritic compounds due to supersaturated Ce atoms being expelled from the solidified grains and accumulated at the front of the interface between solid and liquid during solidification [16]. Hence, the formation and refinement of the AlCuCe phases are promoted through Ce addition.

**Figure 9:**
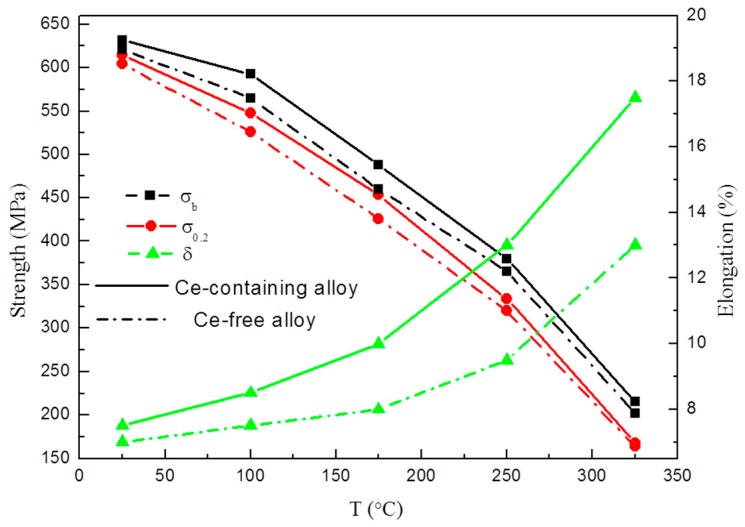
High-temperature tensile curves of Ce-containing and Ce-free alloys at T8 temper.

To study the effects of the refined Ce-containing intermetallic on high-temperature tensile properties of the experimental alloys at T8 temper, the tensile test curves of Ce-containing and Ce-free alloys were investigated at 25 °C, 100 °C, 175 °C, 250 °C, and 325 °C, respectively, as shown in Figure 9. The strength value of Ce-containing alloy is always higher than that of Ce-free alloy within the entire tensile test temperature range. The yield strength of the Ce-containing alloy is higher than that of Ce-free alloy, by 21.5 MPa at 100 °C, by 27.5 MPa at 175 °C, by 13.5 MPa at 250 °C, and by 4.5 MPa at 325 °C. As the temperature increases, the strength difference between the two alloys decreases, whereas the elongation of Ce-containing alloy increased greatly from 7.5% to 17.5% at the testing temperature range between room temperature and 325 °C, while that of Ce-free alloy only increased from 7% to 13%. Ce addition effectively enhances the high-temperature ductility of the Al–Cu–Li alloy, and the strength of this alloy is properly maintained to a certain extent at the same time.

**Figure 10:**
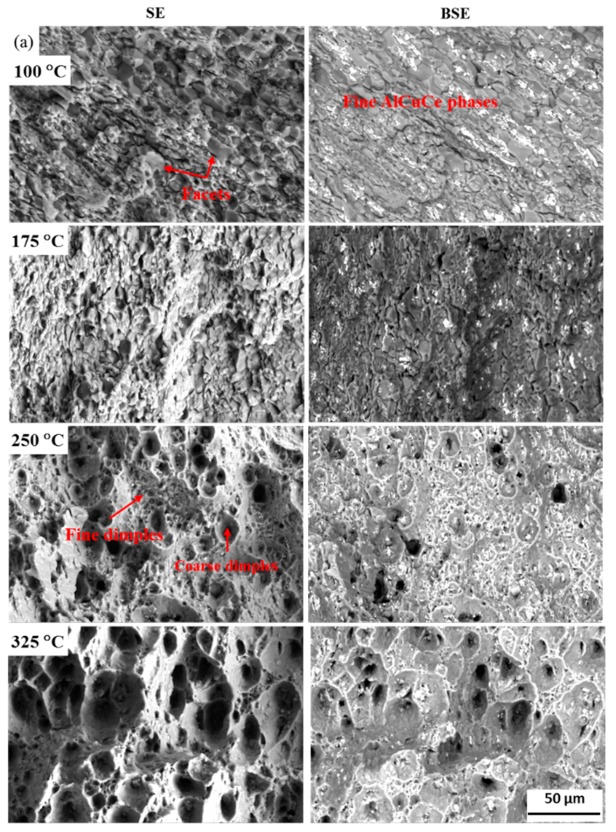

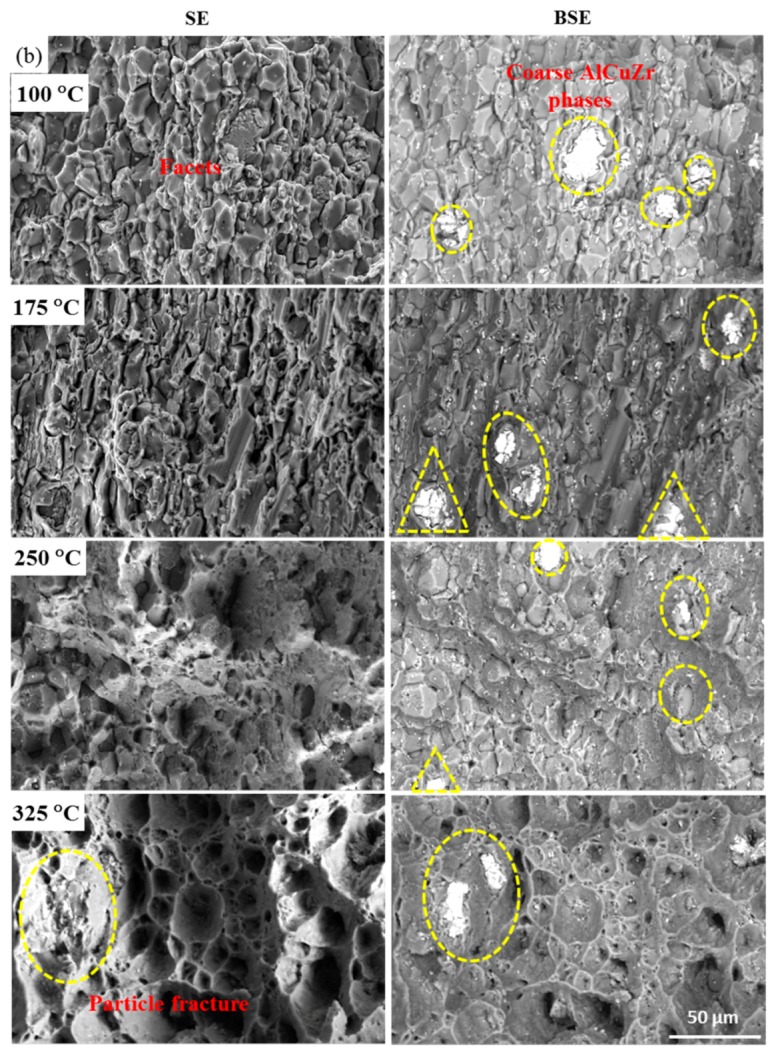
Fracture morphology of (**a**) Ce-containing and (**b**) Ce-free alloys after high-temperature tensile at 100 °C, 175 °C, 250 °C, and 325 °C.

**Figure 11:**
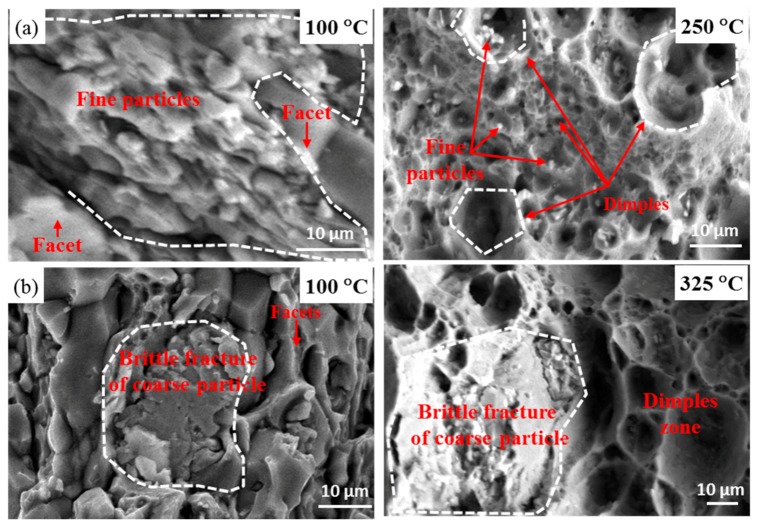
Typical fracture morphologies of (**a**) Ce-containing and (**b**) Ce-free alloys after high temperature tensile at 100 °C, 250 °C, and 325 °C.

To understand the effects of the refined Cu-containing intermetallic particles on the fracture mode of Ce-containing and Ce-free alloys after high temperature tensile, the fracture morphologies of the alloys were investigated comparatively by secondary electron (SE) image and backscatter electron (BSE) image, as shown in Figure 10 and Figure 11. The cleavage facets, which indicate a brittle fracture, are dominant in Ce-free alloy, while only a few facets are seen in Ce-containing alloy at the lower tensile test temperature. Moreover, the fine AlCuCe particles are located in an area with obvious plastic fracture characteristics. As the temperature reaches 250 °C, a large number of dimples are observed in Ce-containing alloy. The dimples in the zone with densely distributed small AlCuCe particles decrease in size and increase in quantity, which is consistent with the obvious increase of the number of dimples in tensile fracture surface of A357 alloy by addition of rare earth element [34]. As the temperature rises to 325 °C, the dimples further increase in size and are distributed more uniformly. The AlCuCe particles become finer and more dispersive because the fine AlCuCe particles are gradually crushed due to the further softening of the alloy matrix at higher temperature [1,35]. Compared with Ce-containing alloy, the coarse AlCuZr particles are prone to brittle fracture and thus would not contribute to the plastic deformation of the alloy at the whole high-temperature tensile test. Therefore, the small high-temperature stable AlCuCe phase in the Ce-containing alloy is beneficial to the improvement of the ductility of the Al–Cu–Li alloy in high-temperature tensile test.

In general, the deformation resistance of metals decreases with temperature. As the temperature increases, the cross-slip and climb of dislocations become easier and the bonding force between atoms is further weakened, which lead to the thermal activation of alloy increase [36]. A large number of studies [37,38,39,40] on the deformation behavior of aluminum alloys at elevated temperatures indicated that intermetallic phases with high-temperature stability have a strong pining effect on grain boundaries and sub-grain boundaries, which can compensate for the grain boundary weakening at high temperatures. A large number of fine rare earth intermetallic phases with high temperature stability lead to improved high temperature tensile properties of the Al alloy, e.g., the tensile strength of 2519 alloy with 0.2 wt% Y increased by 30% at 300 °C, which was attributed to the Al_6_Cu_6_Y phase hindering the movement of grain boundary and the deformation of matrix [41]. The Al_11_La_3_ particles were very stable in Al–Cu–La alloy and widely distributed in grain boundaries or dendritic interstices, which effectively hindered dislocation and grain boundary migration during high temperature creep [14]. A large number of small Al_8_Cu_4_Ce phases on dispersoid-free grain boundaries have been found in our experimental alloy [1]. These high temperature stable fine particles can produce Zener pinning pressure on Zr dispersoid-free grain boundaries, and retard its migration during high-temperature annealing. It is expected that the small Al_8_Cu_4_Ce dispersed phase mainly distributed at grain boundaries can effectively pin dislocations and grain boundaries, which is beneficial to the improvement of high-temperature tensile deformation uniformity and strength of Al–Cu–Li alloy.

## 4. Conclusions

The inhibition of the main strength phase T1 coarsening through 0.29 wt% Ce additions during high-temperature exposure and the effect of element Ce on the diffusion of Cu in Al–Cu–Li alloy were investigated. Moreover, the enhancement of deformation uniformity and strength of the Al–Cu–Li alloy due to refined Al_8_Cu_4_Ce intermetallic phase in the high-temperature tensile test were also studied in this paper. The following conclusions can be drawn.

(1) The thermal stability of Al–Cu–Li alloy is substantiality improved by Ce addition during thermal exposure at the medium-high-temperature stage (170 °C to 270 °C). The enhanced thermal stability of Ce-containing alloy can be attributed to the coarsening inhibition of T1 through impeding the diffusion of Cu. Ce segregates at the interface of the T1 phase can restrict its lateral growth, thereby inhibiting its coarsening.
(2) As the test temperature increases from room temperature to 325 °C, the elongation of Ce-containing alloy increased rapidly from 7.5% to 17.5%, while that of Ce-free alloy only increased from 7% to 13%. The high tensile strength of Ce-containing alloy is always higher than that of Ce-free alloy. The high-temperature stable small Al_8_Cu_4_Ce phase in Ce-containing alloy can effectively pin dislocations and grain boundaries, which is beneficial to the improvement of the ductility and strength of the alloy in the high-temperature tensile test.

## Figures and Tables

**Table 1:** Chemical composition of investigated alloys (wt%).

Alloys	Li	Cu	Ag	Mg	Zr	Ce
Ce-free alloy	1.29	4.62	0.41	0.39	0.14	–
Ce-containing alloy	1.31	4.63	0.38	0.40	0.13	0.29
